# The Utilization of Inert Materials for the Control of Stored-Product Mites—A Mini Review

**DOI:** 10.3390/insects16010078

**Published:** 2025-01-14

**Authors:** Christos G. Athanassiou, Christos I. Rumbos, Paraskevi Agrafioti, Maria K. Sakka

**Affiliations:** 1 Laboratory of Entomology and Agricultural Zoology, Department of Agriculture, Plant Production and Rural Environment, University of Thessaly, Phytokou Str., 38446 Nea Ionia, Magnesia, Greece; athanassiou@uth.gr (C.G.A.); agrafiot@uth.gr (P.A.); msakka@uth.gr (M.K.S.); 2 Department of Agriculture, University of Patras, 30200 Messolonghi, Greece

**Keywords:** diatomaceous earths, inert dusts, non-chemical control, stored-product mites, stored-product protection

## Abstract

Stored-product mites are important pests of stored products, while their presence in storage and processing facilities has a significant effect on public health. On the other hand, inert materials are promising alternatives to conventional pesticides in stored product protection and have provided very good results against storage insects. These formulations can be applied either directly on the product or on surfaces, as dusts or as slurry formulations. In the current paper, we review the factors that affect the efficacy of inert dusts, emphasizing in diatomaceous earths, against stored-product mites. Hence, we address the different biotic and abiotic factors that affect the acaricidal effect of inert materials against different mite species, but also the complexity of such an application, that often arises from the simultaneous presence of plant-feeding mites with their mite predators. Finally, we provide some insights for further research directions.

## Simple Summary

Stored-product mites are significant pests in storage and processing facilities, posing risks to public health. Inert materials, which are alternatives to conventional pesticides, have shown promising results in controlling post-harvest pest infestations, particularly storage insects. These materials can be applied directly to products or surfaces in dust or slurry form. This paper reviews factors that influence the effectiveness of inert dusts, particularly diatomaceous earth, against storage mites. We examine how different biological and environmental factors impact their success, noting the challenges of dealing with both pest mites and their natural predators. Finally, we suggest directions for further research.

## 1. Introduction

Stored-product arthropods can cause serious quantitative losses and considerable qualitative degradations [1]. Indicatively, 10% of the annual production of durable commodities in developed countries is lost due to insect infestations, whereas in developing countries post-harvest losses may reach up to 20% [1]. Most species of economic importance belong to the Order Coleoptera, followed by Lepidoptera and Psocoptera. Nevertheless, there is an emerging pest category that can easily infest different durable commodities: mites (Acari). Most of those species belong to the Order Astigmata, but there are many species that are classified in different other orders, particularly Prostigmata and Mesostigmata [2]. Some of those species have a noticeable significance for public health, as they are directly related to allergenic reactions, skin irritations, etc. [3]. Finally, mites co-exist and co-infest products with stored-product insects, but control measures that are taken against insects are often falsely regarded as effective against mites as well.

Current methods for post-harvest arthropod management mainly rely on chemical control. However, insect resistance development, as well as public concerns for health issues related to pesticide residues in treated commodities, have triggered extensive research on alternative, non-chemical control measures [1]. Inert materials have attracted considerable interest for their activity against storage arthropods [1]. This review will address the utilization of inert materials for the control of stored-product mites at the post-harvest stages of durable agricultural commodities. This topic was selected for review, given that the most recent work available was published more than twenty years ago by Cook [4] and considering the potential of inert materials for the control of stored-product arthropods, as well as the utilization of these substances as main control measures, especially after the withdrawal of many active ingredients that were until recently in use.

## 2. Importance of Stored-Product Mites

While numerous studies focus on the importance of stored-product insects, there are disproportionally fewer studies for stored-product mites, despite the fact that both insects and mites coexist in durable agricultural commodities [1]. As noted above, the most important stored-product mites are those that belong to Astigmata, especially in the family Acaridae. Different surveys indicate that the most abundant mite species in grains and related products, i.e., flour, bran, etc., are the cheese mite, *Tyrophagus putrescentiae* (Schrank) (Acaridae), and the flour mite, *Acarus siro* L. (Acaridae) [5,6]. Nevertheless, there are numerous other species with considerable occurrence in different commodities, such as *Carpoglyphus lactis* (L.) (Glycyphagidae), which is often recorded in dried fruits and other saccharide-rich substrates [7]. Interestingly, Astigmata often coexist with other mites that are predators, to a much larger extent than insects coexist with their predators, suggesting the unique fauna that mites can form in a very short time because of the shorter developmental time of mites than insects. Some examples are *Blattisocius tarsalis* (Berlese) (Mesostigmata: Blattisociidae), and to a lesser extent, the relative *Blattisocius keegani* Fox, which is often found together with *B. tarsalis* in the same facilities [2,5,8]. These species are predators of other stored-product mites, but can also feed upon the eggs of stored-product moth species, particularly those of the family Pyralidae [2,8,9]. Another example of frequently detected mite species in storage and processing facilities is some Prostigmata of the family Cheyletidae, such as *Cheyletus eruditus* (Schrank) and its relative *Cheyletus malaccensis* Oudemans, which seems to be more adapted to drier and warmer climatic zones [5,10]. Those are important predators of both mites and insects, and, in fact, *C. eruditus* has been commercialized as a predatory mite formulation for augmentative releases in biocontrol-based strategies [11,12].

Stored-product mites can cause damage at different levels. Hubert [2] classifies these infestations into three broad categories: a) the direct infestation of different commodities through feeding; b) symbiosis with different other groups of organisms, such as fungi and bacteria, that can be further indirectly introduced into the product and cause serious degradation, which may render the commodity unsuitable as food and feed; and c) the production of different compounds that can be directly hazardous such as allergens. Mites are also very common in the urban environment, in houses, hospitals, etc., and this is why they have largely been characterized as “synanthropic” [3,13]. When the conditions are suitable for their development, stored-product mites can easily build extremely high population densities in a very short time. Collins [14] reported that high relative humidity and/or moisture content values are of utmost importance for the development of most stored-product mites, but some species, such as *T. putrescentiae*, can also develop in drier areas [15]. Regarding temperature, mites are more adapted to lower temperatures than stored-product insects and can survive at temperatures lower than 10 °C [14], thus aeration or even grain cooling may not be effective [16]. Moreover, mites can continue to infest the product even during wintertime, while insects may not be active during that period [5,8].

One other characteristic that should be taken into account is the fact that mites can remain undetectable for a long time, due to their small size, and their presence can be revealed when the infestation is too great [2]. In this context, even regular sampling may not be effective in estimating mite densities on grains and other commodities, since this estimation is highly laborious and requires specialized personnel and certain equipment, such as a Berlese–Tullgren funnel device [5]. Moreover, many mite species have been proven to be tolerant to some of the common insecticides that are currently in use in stored-product protection. For instance, Hasan et al. [17] found that *T. putrescentiae* individuals were tolerant to the fumigant sulfuryl fluoride, with eggs of this species to be the most tolerant life stage. Similar results have been reported in the case of phosphine, where *T. putrescentiae* was able to survive concentrations that are usually effective for the control of stored-product insects [18]. Furthermore, Sánchez-Ramos and Castañera [19] found that the bacteria-based pesticide spinosad, which is now registered as a grain protectant, was unable to control *T. putrescentiae*, even at extremely high dose rates.

Based on the above and considering the inability of many of the conventional pesticides to provide sufficient mite control, along with the withdrawal of many active ingredients, it is essential to evaluate different active ingredients and control methods that can be used with success for the control of these notorious pests. Among these, inert materials seem to play an important role in moving in this direction.

## 3. Diatomaceous Earths and Other Inert Materials

Inert materials that are used in stored-product protection can be classified into four broad categories [20,21,22]. The first category is the non-silicaceous materials, such as lime, katelsous (rock phosphate and ground sulfur), and salt, while the second is different types of soil-related materials, such as sand, kaolin, and clay. These two groups of inert materials have limited use and are mostly applied by farmers in developing countries. The third category includes a series of minerals and natural deposits that are rich in silicon dioxide, such as diatomaceous earths (DEs) and zeolites, which have been extensively evaluated in stored product protection. Finally, the fourth category includes the different types of synthetic silicates, such as silica aerogels, which have also been evaluated at the post-harvest stages of different agricultural commodities.

The mode of action of these materials is purely mechanic, since they act through the cuticle, causing death through water loss, while other secondary modes of action may have an effect in parallel, such as a repulsive activity [22,23]. Different inert dusts, such as DEs, have an extremely low or no mammalian toxicity, which makes them ideal as alternatives to conventional pesticides, such as organophosphorous compounds and pyrethroids [21]. They are effective as grain protectants, i.e., as an admixture with grains, but can also be effective when applied on different surfaces [22]. To be effective, these dusts should be applied at high applications, usually 1000 ppm (1 g/kg of grain) or even higher [23], but they can be easily removed from the commodity [24]. At the same time, their presence does not affect the final product [25].

Currently, there are several commercially available formulations of DEs, and, to a lesser extent, of other inert dusts, that are used in stored-product protection, especially in organic products for which the application of the conventional contact insecticides and fumigants is not an option. Nevertheless, most of the data available so far are focused on the use of these materials against insects, while data are scarce for their effect on stored-product mites. This is exactly the purpose of the current work: to gather all the published data available so far for the control of stored-product mites, and the factors that should be taken into account when an inert dust-based strategy is planned. As most of the publications that examine this topic are focused on DEs, we will mostly use DEs as an example of the utilization of silicaceous materials for the control of stored-product mites.

## 4. The Effect of Abiotic Factors on the Efficacy of Inert Materials Against Storage Mites

The effect of the different abiotic factors, such as temperature and relative humidity/moisture content, is binary: directly, through the activity of these compounds, and indirectly, through the behavior of the target species in the treated substrate. The effect of temperature has been extensively tested in the case of different stored product insect species when they are exposed to DE-treated commodities, especially grains [9,23,26,27]. For instance, Athanassiou et al. [27] found that an increase in temperature increased DE efficacy against the adults of the rice weevil, *Sitophilus oryzae* (L.) (Coleoptera: Curculionidae), and the confused flour beetle, *Tribolium confusum* Jacquelin du Val (Coleoptera: Tenebrionidae). In that study, the authors postulated that this effect was due to the fact that an increased temperature increases water loss and insect stress, while insect movement is more pronounced at higher temperatures [27]. Similar results have also been reported in the case of stored-product mites by Palyvos et al. [15]. In that study, the authors reported that the mortality of *T. putrescentiae* was higher at 25 than at 20 °C, regardless of the grain commodity on which the DE was applied. Moreover, Athanassiou and Palyvos [9] found that the mortality of *B. keegani* was higher at 30 °C than at 20 or 25 °C on DE-treated grains. This was expected, considering the susceptibility of stored-product mites at high temperatures [14]. In addition, the combinations of heat treatments with DEs have been already proposed as a viable alternative to structural treatments at a commercial scale, given that DEs will further enhance the water stress that is caused by heat [28,29].

The increase in humidity/moisture seems to have a negative effect in DE efficacy against stored-product insects and mites [14,22,23]. Obviously, the increase in humidity/moisture has a detrimental effect on the DE particles, making them less effective [21,23]. In addition, insects and mites can moderate water loss better under moist conditions. Nevertheless, studies on the effect of humidity/moisture on insects and mites that are exposed on DE-treated substrates are often contradictory, but in general, most studies concur that mortality is higher in drier conditions [14,26,30,31]. For instance, Cook and Armitage [30] found that, on grains with a moisture content of 16%, all mites were dead after 2 weeks, while complete mortality took 5 weeks on grains that had a 17% moisture content, suggesting that even the slightest increase in moisture content can drastically reduce the efficacy of DEs. However, in some stored-product insects it has been reported that some DE formulations were not much affected in their efficacy by the level of humidity/moisture [26]. The “speed of kill” is an essential parameter especially on grains that have high moisture content values, as mites can harbor fungi and other pathogens that may produce different types of toxins on the grains [3], making the damage irreversible.

DEs can also be used against stored-product mites as a surface treatment, as was tested in the series of bioassays that were carried out on wooden surfaces by Collins and Cook [32]. In that work, it was found that the slurry treatments were much less effective than the dust treatments against the cosmopolitan food mite, *Lepidoglyphus destructor* (Schrank) (Astigmata: Glycyphagidae), and also against the granary weevil, *Sitophilus granarius* (L.) (Coleoptera: Curculionidae), and the Mediterranean flour moth, *Ephestia kuehniella* Zeller (Lepidoptera: Pyralidae). Kilic [33] tested local DEs from Turkey and found that the application of both wettable powder and dust formulations was equally effective for the control of *T. putrescentiae*.

## 5. The Effect of Biotic Factors on the Efficacy of Inert Materials Against Storage Mites

One of the most important biotic factors that should be considered in the application of DEs and other inert dusts is the target species. Palyvos et al. [15] reported that *T. putrescentiae* was very susceptible to DE-treated grains, given that complete mortality of this species occurred within 48 h of exposure, while even at the 24 h exposure interval, the mortality of the exposed individuals was extremely high. Moreover, this high efficacy was recorded regardless of the grain that was treated with DEs [15]. It is generally considered that mites, as soft-bodied, are highly susceptible to silicaceous materials, and that desiccation occurs in a very short time, which is usually shorter than the time that is required for stored-product insects. Of the insect species tested so far, the rusty grain beetle, *Cryptolestes ferrugineus* (Stephens) (Coleoptera: Laemophloeidae), is considered to be highly vulnerable to DEs, due to the “thin” shape of its body, which accelerates water loss [23,24]. Nevertheless, this is not the case for other soft-bodied stored-product insects, such as psocids, that have an internal mechanism to moderate water loss after exposure to DEs [34]. Hence, the susceptibility classification of the different mite species according to their body size and shape is rather simplistic and should be avoided, although some general conclusions can be drawn.

From the data available so far, it seems that *A. siro* is less susceptible than *T. putrescentiae*, although the comparable data are extremely scarce [14,15,30,32,35]. In an earlier work, Cook and Armitage [30] found that *A. siro* can survive for more than a week on DE-treated grains, an interval that was much higher than the one reported for *T. putrescentiae* [15]. Still, in general, both species are considered very susceptible to DEs, at concentrations that are 1000 ppm or even lower. Similarly, DEs were found able to suppress house dust mite (Pyroglyphidae) populations and the expression of allergens within a very short time [36].

There are mite species that are considered as tolerant to DEs, in contrast to *A. siro* and *T. putrescentiae*. For example, *L. destructor*, which is also an important mite in public health terms, has been found to be equally susceptible to DEs as certain stored-product insect species, such as *S. granarius* and *E. kuehniella* [32]. Moreover, some of the predatory mites were much less susceptible than *A. siro* and *T. putrescentiae* [9,15]. For example, Palyvos et al. [15] found that, in the same DE-treated substrate, the predator *C. malaccensis* was by far less susceptible than its prey, *T. putrescentiae*. In fact, in that study, the authors reported that the mortality of *C. malaccensis* was even lower when the prey was present, which could be attributed to the fact that feeding could moderate the adverse effects of the DE particles [15], e.g., through the restricted movement of the predator due to feeding that results from reduced DE exposure. Moreover, it has been hypothesized that the feeding and movement behavior of these two species are qualitatively different, as *T. putrescentiae* is in direct contact with the treated substrate, while *C. malaccensis* has a prey-seeking behavior that may not be related with the treated grain per se [15]. The tolerance of predatory mites has also been confirmed in a follow-up study by Athanassiou and Palyvos [9], where it was reported that the mortality of both *B. keegani* and *C. malaccensis* was lower compared to the mortality levels that had been recorded for *T. putrescentiae* by the Palyvos et al. [15] study. This is probably linked with the movement behavior that was mentioned above, but also with the fact that the exoskeletons of both Mesostigmata and Prostigmata are thicker than that of Astigmata. In addition, the respiration of Mesostigmata and Prostigmata is carried out through tracheae, while Astigmata have only dermal respiration, which may increase stress through water loss [9]. Additional experimental work is warranted to evaluate this hypothesis.

The life stage of the target mite species is also a determinative factor that affects the efficacy of inert materials. For instance, the protonymphs of both *B. keegani* and *C. malaccensis* were proven to be less susceptible than adults to DEs [9], which could be attributed to the fact that protonymphs can undergo ecdysis and can thus partially replace the damaged exoskeleton. However, this trend was related to temperature, given that the classification of the tolerance of protonymphs and adults was temperature-mediated, probably due to the fact that some life stages are more susceptible to heat than others [9]. In another study, Iatrou et al. [35] found that the adults of *T. putrescentiae* were more tolerant than immature stages, and that the mortality of this species was high, even at dose rates as low as 200 ppm. Although there are no indications that DEs have an ovicidal effect [23], increased immature control can eventually lead to population suppression.

## 6. Other Factors that Affect the Efficacy of Inert Materials Against Storage Mites

The type of commodity to which the inert materials are applied is another crucial factor that considerably affects their performance against storage mites. Some studies underline the importance of the commodity for DE efficacy, and that DEs are less effective on some commodities compared to others [22,37]. For instance, Kavallieratos et al. [37] found that DEs are less effective on maize than on wheat and other small grains, due to differences in the adherences of the DE particles. However, there are only a few studies that have examined the effect of commodity on the efficacy of different DEs against stored-product mites. Palyvos et al. [15] tested DEs against *T. putrescentiae* on wheat, maize, oats, and rye, and found no considerable differences among the different commodities, probably due to the fact that mite mortality was extremely high in a very short time. Still, in that study, the authors identified some differences in the mortality of *C. malaccensis* that were commodity-related, and in many of the combinations tested, mortality on maize was lower than the corresponding levels of the other grains [15]. Table 1 summarizes the most important and relevant research works with inert materials and mites associated with stored durable commodities.

## 7. Key Point Summary and Prospects for the Future

Apart from DEs, other inert materials have also been proven to be promising against stored-product mites, such as zeolites. For instance, three commercial zeolite formulations provided significant control against different life stages of *A. siro* and *T. putrescentiae* [38]. However, the data that are currently available for different types of inert materials, other than DEs, for mites that infest stored products are extremely limited. Therefore, more research is warranted on the use of inert dusts, especially on non-DE inert materials, for the control of storage mites. More specifically, we consider that work with inert materials for the control of stored-product mites should be focused on three major areas:

i. There are not that many data available on the efficacy of these dusts on different surfaces, given that many contact insecticides that are currently in use in storage and processing facilities have different efficacy levels on the different surfaces that they are applied to [39,40].

ii. The combination of inert dusts with other substances may be even more effective against mites, which is something that has to be tested more thoroughly. Previous studies have shown the value of the combined effect of inert dusts with other substances, such as entomopathogenic fungi and natural insecticides [22].

iii. Additional work is needed to further reduce the required dose rates of inert materials for stored-product mite control, given that the admixture of inert materials with grain can have adverse effects on some of its properties, especially on its bulk density [24].

Considering the above parameters, and also the need for judicious pest control methods, inert materials can play a considerable role in stored-product protection as acaricides. Taking into account the consumers’ demand for residue-free food, and also the fact that the plant protection products (PPPs) that are applied at the post-harvest stages of agricultural commodities are the closest PPPs to food, it becomes evident that inert materials offer a viable nontoxic residue-free solution that can be adaptable in many key application scenarios.

## Figures and Tables

**Table 1:** Inert materials that have been tested with mites associated with stored durable commodities.

Suborder	Family	Mite Species	Life Stage	Commodity/Surface	Inert Material		Reference
Astigmata	Acaridae	*Tyrophagus putrescentiae*	adults	hard wheat oats rye maize	diatomaceous earth	SilicoSec (92% SiO_2_; 8–12 μm particle size (PS))	[15]
			adults	concrete surface	diatomaceous earth	Detech^®^ WP95; Detech^®^ Dust (80.6% SiO_2_; median PS = 14 μm)	[33]
			adults nymphs larvae	hard wheat	diatomaceous earth	SilicoSec (92% SiO_2_; 8–12 μm PS) PyriSec (95.7% SilicoSec; 8–12 μm PS) Insecto (86.7% SiO_2_; 0.8 and 52 μ PS) Protect-It (83.7% SiO_2_; 5–6 μm PS) DEA-P (DE (83% DE with 89% SiO_2_; median PS = 10 μm)) and abamectin (0.25%)	[35]
			larvae nymphs adults	hard wheat	zeolite	Raw zeolite (0–1000 μm PS) Zeofeed (0–1000 μm PS) Zeoprofeed Land 93 (0–800 μm PS)	[38]
		*Acarus siro*	mixed stage	wheat	diatomaceous earth	Dryacide	[30]
			larvae, nymphs adults	hard wheat	zeolite	Raw zeolite (0–1000 μm PS) Zeofeed (0–1000 μm PS) Zeoprofeed Land 93 (0–800 μm PS)	[38]
	Glycyphagidae	*Lepidoglyphus destructor* (syn. *Glycyphagus destructor*)	mixed stage	wheat	diatomaceous earth	Dryacide	[30]
			adults	wooden surface	diatomaceous earth	SilicoSec (92% SiO_2_; 8–12 μm PS) Diasecticide^TM^ (83.7% SiO_2_)	[32]
Mesostigmata	Ascidae	*Blattisocius keegani*	protonymphs adult females	hard wheat	diatomaceous earth	SilicoSec (92% SiO_2_; 8–12 μm PS) PyriSec (95.7% SilicoSec; 8–12 μm PS)	[9]
Prostigmata	Cheyletidae	*Cheyletus malaccensis*	protonymphs adult females	hard wheat	diatomaceous earth	SilicoSec (92% SiO_2_; 8–12 μm PS) PyriSec (95.7% SilicoSec; 8–12 μm PS)	[9]
			adults	hard wheat oats rye maize	diatomaceous earth	SilicoSec (92% SiO_2_; 8–12 μm PS)	[15]

## Data Availability

No new data were created.
